# Affix polyfunctionality in French deverbal nominalizations

**DOI:** 10.1007/s11525-022-09401-4

**Published:** 2022-12-19

**Authors:** Justine Salvadori, Richard Huyghe

**Affiliations:** University of Fribourg, Av. de l’Europe 20, Fribourg, 1700 Switzerland

**Keywords:** Derivation, Affix, Polyfunctionality, Nominalization, French

## Abstract

This article investigates the semantic polyfunctionality of affixes, i.e. their ability to serve a variety of distinct semantic functions. Based on the analysis of a sample of 3,091 deverbal nouns ending with 46 different suffixes in French, the study examines the diversity of semantic functions realized by deverbal suffixes, the distribution of these functions across suffixes and the relationships that may exist between different functions. It appears that polyfunctionality is widespread among French deverbal suffixes and involves a large number of semantic functions, with highly variable realization frequency. Several fundamental aspects of affix polyfunctionality are further highlighted. A probabilistic analysis shows that polyfunctionality can be driven by non-arbitrary semantic associations between functions. A hierarchy of functions can also be postulated depending on whether they can be instantiated by monosemous or only polysemous derivatives. In addition, polyfunctionality appears to be inseparable from rivalry relationships and to determine the degree of rivalry between affixes. Overall, the study illustrates that affix polyfunctionality is governed by systematic organizing principles whose ramifications touch on lexical ambiguity and morphological competition.

## Introduction

Affixes in the world’s languages are known to be frequently polyfunctional, i.e. the same affix can be used in various morphological operations.^1^ Functions may extend to different lexical classes of bases and derivatives, as in the case of the French suffix -*ment*, which can derive adverbs from adjectives (*fort* ‘loud’ → *fortement* ‘loudly’), or nouns from verbs (*licencier* ‘dismiss’ → *licenciement* ‘dismissal’). Polyfunctionality can also be observed without variation in input and output lexical classes. For example, Kagan (2015) identifies five functions for the prefix *pere-* in Russian, which attaches to verbs and provides additional information about crossing, redoing, excess, comparison or spending time (see also Švedova, 1982; Zinova, 2021). The fact that affixes may serve several functions is hardly surprising. The limited number of affixes in a given language compared to the wide range of semantic functions associated with derivation necessarily leads to affix polyfunctionality. However, the number of functions associated with an affix can be quite variable, and it can be asked whether semantic functions are arbitrarily distributed among affixes, or whether there is a systematic organization of affix polyfunctionality in natural languages.

Affix “polysemy”, as some authors also call it (see e.g. Lehrer, 2003; Lieber, 2004), finds its counterpart in what others refer to as affix “synonymy” (see e.g. Zwanenburg, 2000; Raffelsiefen, 2010). In the latter case, the one-to-many relationship is reversed: No longer is the emphasis on a single affix associated with several functions, but rather on a single function potentially realized by several affixes (Plag et al., 2023). For example, the suffixes *-ão*, *-(i)ano*, *-eiro*, *-enho*, *-ense*, -*ês*, -*ino*, -*o*, -*ita*, -*ota* and -*ol* can all derive gentilic adjectives in Portuguese (Pöll, 2016). In the perspective of system economy, shared functionality is less expected than polyfunctionality. However, a possible motivation for shared functionality could be the existence of conceptual relationships between the different functions of affixes: If two given affixes each serve a distinct function, and the two functions are semantically associated with an identical third function, then the two suffixes will inevitably compete for the third function. Ultimately, the question is raised as to how multiple and shared functions are organized together.

In this paper, we investigate the structures of affix polyfunctionality, focusing on the suffixes that are used to form deverbal nouns in French. These suffixes are known to be abundant and associated with many different semantic outputs, thus constituting an interesting case for the study of affix polyfunctionality. Although French deverbal suffixes have been studied by many authors, the exact extent of their semantic functions remains uncertain. Systematic and detailed accounts of verb-to-noun derivation have been proposed for English (e.g. Lieber, 2016), but no study of this scope has been conducted for French. Among the issues we address are the variety of functions realized by deverbal suffixes, the distribution of these functions across suffixes, and associations that may exist between different functions. Polyfunctionality varies according to the number of functions per affix and their frequency of instantiation in derivatives. We will focus on the former aspect, with the aim of describing as exhaustively as possible the variety of semantic functions served by each deverbal suffix. Nevertheless, we will also provide insights into realization frequency and describe some aspects of affix rivalry, given its close ties with polyfunctionality.

The article is organized as follows. In Sect. 2, we propose a definition of polyfunctionality and review some of the theoretical problems it raises. Sections 3 and 4 introduce the method we followed to collect and analyze data, including principles of semantic description. We present the results in Sect. 5, and report on a case study that addresses the frequency of instantiation of functions in Sect. 6. Overall, our results allow for a better understanding of the semantic operations associated with derivational processes, and of the complex relationships that exist between affix polyfunctionality and rivalry.

## Affix polyfunctionality in question

While many word formation processes exhibit polyfunctionality (e.g. conversion in Germanic and Romance, root-and-pattern in Semitic), the present study focuses on affixation. In this section, we first discuss general aspects of affix polyfunctionality, namely its definition (2.1) and different dimensions (2.2), before introducing the topic of polyfunctionality of deverbal suffixes in contemporary French (2.3).

### Defining polyfunctionality

There is no consensus in the literature on what polyfunctionality entails. The broadest definition is given by Zwanenburg (2000), for whom “polyfunctionality consists in the fact that one affix can serve to form different semantic types of words, and inversely different affixes can serve to form one and the same semantic type of words” (p. 842). A similar stance is taken by Schulte (2015b), who relates polyfunctionality to the fact that “the same affix can often be found in derivatives with a range of meanings, but different affixes also seem to give rise to very similar derivatives” (p. 17). Polyfunctionality is considered here a many-to-many relation rather than a one-to-many relation, although Zwanenburg (2000) concedes that the expression “morphological asymmetry”, borrowed from Karcevskij (1929) and Beard (1984), might be more appropriate for the many-to-many scenario. A more restricted definition is provided by Booij and Audring (2020), who consider polyfunctionality as mainly related to the grammatical diversity of inputs. According to them, it refers to “the ability [of affixes] to attach to bases of different lexical categories” and should not be equated with affix polysemy, which applies when affixes “have more than one meaning”. However, what Booij and Audring (2020) refer to as “polysemy” is called “polyfunctionality” by others, such as Moortgat and van der Hulst (1981) who show that Dutch suffixes can give rise to several semantic subcategories of derivatives for a given part of speech. Prćić (2019) follows the same theoretical line, stating that affixes are polyfunctional when “several distinct, related or unrelated, meanings and distinct functions coincide in a single form” (p. 158).

We will adopt this last definition of polyfunctionality,^2^ avoiding reference to polysemy as a precaution. Not only does the notion of affix polysemy entail the debatable assumption that affixes have a semantic content (Lehrer, 2000; Lieber, 2004; Rainer et al., 2014), but it also presupposes that a semantic link unites two or more functions of a given affix. While some authors explicitly argue for the existence of such links (see e.g. Panther & Thornburg, 2009 for the suffix *-er* in English), or place themselves in theoretical models that suggest relations between functions (see e.g. Plag et al., 2018 in a frame-semantic approach), more often than not, the idea of multiplicity seems to take precedence over semantic relations in analyses. In this work, relations between functions are not postulated, one of the objectives being precisely to probe their existence. One would be hard-pressed to find, for example, a link between some semantic types of output produced by the suffix -*eur*, such as eventualities (*clamer* ‘proclaim’ → *clameur* ‘clamor’), cognitive entities (*valoir* ‘be worth’ → *valeur* ‘value’) and natural substances (*suer* ‘sweat’ → *sueur* ‘sweat’). Provided that affixes have a semantic content, such cases would probably fall under homonymy rather than polysemy.^3^

### Dimensions of polyfunctionality

Affix polyfunctionality is a complex property that varies according to:

- the number of semantic functions associated with an affix;
- the frequency with which these functions are realized;
- the co-realization of functions in ambiguous derivatives.

As mentioned earlier, a first distinction has to be made between the number of functions allowed by a given affix on the one hand, and the frequency of instantiation of these functions on the other. The English suffix -*er*, for example, is considered highly polyfunctional in the literature. According to Bauer et al. (2013), it can give rise to nouns denoting agents (*writer*), experiencers (*hearer*), stimuli (*pleaser*), instruments (*amplifier*), patients (*scratcher*), locations (*smoker*), measures (*fiver*) and inhabitants (*New Yorker*). It is however generally accepted that some of these functions (e.g. location, patient) are relatively marginal compared to others (e.g. agent, instrument), a distinction that seems to be based primarily on their frequency among derivatives. Interestingly, such differences between variety and frequency may play a role in affix rivalry. One can easily imagine that affixes that share all or most of their functions in addition to realizing them at an equal frequency will constitute very strong rivals from a semantic perspective. Conversely, if two given affixes share only part of their functions, or share all their functions but realize them at very different frequencies, their rivalry will be weaker. The existence of different degrees of semantic rivalry, taking into account both levels of analysis, could thus be considered.

Another variable feature of affix polyfunctionality relates to multiplicity of meaning in derivatives. Affix polyfunctionality should be carefully distinguished from lexical ambiguity: The fact that an affix is polyfunctional does not entail that a derivative instantiates its different functions. An affix *α* with two semantic functions A and B is not necessarily used to form ambiguous words with A and B meanings, and the proportion of *α*-derivatives with both meanings can be highly variable. Accordingly, the propensity to form ambiguous derivatives can be considered a feature of affix polyfunctionality, that varies depending on both the functions that are co-realized and the frequency of these co-realizations.

The identification of affix polyfunctionality in ambiguous derivatives can be problematic. While it is well known that derived words are frequently ambiguous (see e.g. Rainer, 1996), the exact origin of their ambiguity is often difficult to pinpoint, especially when the different meanings of a derivative are related to the same meaning of the base. Different morphosemantic configurations are theoretically conceivable. Lexical ambiguity may result from the existence of multiple word-formation patterns associated with the same affix, and therefore directly depend on the versatility of morphological processes. From this perspective, Ferret and Villoing (2015) argue that the instrument reading of French event nominalizations in -*age* results from a morphological rule conditioned by specific constraints on base verb properties. However, the ambiguity of derivatives could also result from figurative semantic extensions (metonymic or metaphorical in nature) that are independent of morphological processes. Bauer (2017) recognizes in this regard that one should leave open “the possibility that sometimes the metonymy affects the whole word and not just the affix” (p. 6).

Even on the assumption that a semantic extension is by nature metonymic or metaphorical, the formation of ambiguous nouns could still be a property of the affix. The existence of a complex derivational pattern including a figurative component could be established for a given affix if it appears that (i) a semantic category is present only in ambiguous derivatives and (ii) the association of semantic categories is not generalized across comparable affixes. In French, for example, some polysemous derivatives in -*ion* denoting events sometimes have another meaning that refers to groups of agents (e.g. *immigration* ‘immigration’/‘migrants’, *inspection* ‘inspection’/‘inspectorate’, *rédaction* ‘writing’/‘editorial board’, *rébellion* ‘rebellion’/‘rebels’). This second interpretation actually never appears among monosemous derivatives in *-ion* and cannot be associated with rival suffixes *-age*, -*ure*, -*ade*, -*ance* and *-aison*. The formation of word meanings that fit into a regular polysemy pattern could be seen as a subsidiary property of the suffix and therefore be investigated as part of affix polyfunctionality.

### The polyfunctionality of French deverbal suffixes

Affix polyfunctionality appears to be widespread across the world’s languages. As an indication, 26 of the 55 languages listed in Štekauer et al. (2012)’s sample have affixes with one-to-many relations. Despite this cross-linguistic representation, polyfunctionality is unevenly addressed in the literature. On the one hand, not all languages have received the same attention over the years, many studies focusing on Indo-European languages (Štekauer et al., 2012, p. 171). On the other hand, suffix polyfunctionality seems to be more frequently examined than prefix polyfunctionality, which may be explained by certain characteristics of suffixes. For example, in the case of verb-to-noun suffixation, the great variety of semantic functions as well as the large number of suffixes involved call for investigation. Despite the numerous studies that have been devoted to particular suffixes (see e.g. Booij, 1986 for *-er* in Dutch; von Heusinger, 2005 for *-ata* in Italian; Müller, 2011 for *-er* in German; Kawaletz & Plag, 2015 for *-ment* in English) or groups of suffixes (see e.g. Fábregas, 2010 for -*ción* and -*miento* in Spanish; Schulte, 2015a for *-age* and *-ery* in English; Varvara, 2020 for *-mento* and *-zione* in Italian), the subject is far from being exhausted. Detailed comprehensive studies of the polyfunctionality of suffixes in a given language are still very few.

As far as French is concerned, many researchers have investigated the versatility of one or several suffixes, often with an interest in morphological competition, and especially in the case of verb-to-noun derivation (see e.g. Uth, 2008 for *-age* and *-ment*; Ferret et al., 2010 for *-ée* and *-age*; Martin, 2010 for *-age*, *-ion* and *-ment*). However, some gaps can be observed. In spite of the generous literature already available for some suffixes (see e.g. Aliquot-Suengas, 2003 for *-ade*; Plénat, 1999 for *-aille*; Ferret & Villoing, 2015 for *-age*; Schnedecker & Aleksandrova, 2016 for *-aire*; Burdy, 2013 for *-aison*; Dal & Namer, 2010 for *-ance*; Plénat, 2005 for *-ette*; Huyghe & Tribout, 2015 for *-eur*; Vendryes, 1946 for *-is*; Villoing & Namer, 2008 for *-oir*; Zellmer, 1935 for *-ure*), few authors seek in principle to describe the polyfunctionality of deverbal suffixes in their entirety. The focus is often on one or a few specific functions, such as the formation of nouns denoting humans (e.g. Schnedecker & Aleksandrova, 2016), instruments (e.g. Ferret & Villoing, 2015), means (e.g. Fradin, 2012), or events (e.g. Martin, 2010). Moreover, semantic classifications used to categorize French derivatives are often rudimentary and questionable with respect to their lexical coverage. The present study aims to fill these gaps by proposing a systematic and global investigation of the polyfunctionality of all potential deverbal suffixes, using a detailed classification for the semantic description of derivatives.

## Data collection

This section presents the method we used to collect the data analyzed in the study. We first list the different suffixes used to derive nouns from verbs in French (3.1), before turning to the selection of a sample of deverbal nouns (3.2).

### Deverbal suffixes

The present study is based on the investigation of 46 suffixes presented in Table 1. While most of these suffixes are mentioned in general studies dedicated to word formation in French (Dubois, 1962; Apothéloz, 2002; Thiele, 1987), we also identified a few complementary suffixes that are sometimes not considered deverbal. For example, although Thiele (1987) states that *-ase* can only select nominal bases, it seems difficult not to associate in synchrony a verbal base to nouns such as *invertase* ‘invertase’ (← *inverser* ‘reverse’) and *réductase* ‘reductase’ (← *réduire* ‘reduce’). In addition, potentially allomorphic suffixes were treated separately if they differed in gender, as we do not postulate semantic equivalence between morphological patterns of gender-varying forms. This distinction concerns: -*eur*, -*eresse*, -*eure*, -*euse* and -*rice*; *-ant/-ent* and *-ante/-ente*; *-ard* and *-arde*; *-eau* and *-elle*; *-er/ier* and *-ère/ière*; *-et* and *-ette*; *-in* and *-ine*; *-oir/-oire*^4^ and *-oire*; *-on* and *-onne*. Considering gender variation as a sufficient condition for suffix distinction, we also distinguished a priori two homographs -*aire* and -*iste* forming masculine and feminine nouns, even if they seem to vary in gender only with respect to human denotation. In the absence of gender variation, orthographic variants were analyzed together (e.g. -*ance*/*ence*, -*ote*/*otte*).

**Table 1:** List of deverbal suffixes with examples

Suffix	Derived noun	Base verb
-*ade*	*embrassade* ‘kissing’	*embrasser* ‘kiss’
-*age*	*décapage* ‘scouring’	*décaper* ‘scour’
-*ail*	*éventail* ‘fan’	*éventer* ‘fan’
-*aille*	*bataille* ‘battle’	*se battre* ‘fight’
-*ain*	*levain* ‘leaven’	*lever* ‘rise’
-*aire* masc.	*commentaire* ‘commentary’	*commenter* ‘comment’
-*aire* fem.	*locataire* ‘tenant’	*louer* ‘rent’
-*aison*	*démangeaison* ‘itching’	*démanger* ‘itch’
-*ance*/-*ence*	*espérance* ‘hope’	*espérer* ‘hope’
-*ant*/-*ent*	*fabricant* ‘maker’	*fabriquer* ‘make’
-*ante*/-*ente*	*imprimante* ‘printer’	*imprimer* ‘print’
-*ard*	*scribouillard* ‘penpusher’	*scribouiller* ‘scribble’
-*arde*	*dégonflarde* ‘chicken’	*se dégonfler* ‘chicken out’
-*ase*	*réductase* ‘reductase’	*réduire* ‘reduce’
-*asse*	*tétasse* ‘breast’	*téter* ‘suckle’
-*eau*	*traîneau* ‘sledge’	*traîner* ‘drag’
-*elle*	*sauterelle* ‘grasshopper’	*sauter* ‘jump’
-*er*/-*ier*	*braconnier* ‘poacher’	*braconner* ‘poach’
-*ère*/-*ière*	*pissotière* ‘public urinal’	*pissoter* ‘urinate’
-*eresse*	*pécheresse* ‘sinner’	*pécher* ‘sin’
-*erie*	*brasserie* ‘brewery’	*brasser* ‘brew’
-*et*	*couperet* ‘chopper’	*couper* ‘chop’
-*ette*	*zappette* ‘zapper’	*zapper* ‘zap’
-*eur* masc.	*réfrigérateur* ‘refrigerator’	*réfrigérer* ‘refrigerate’
-*eur* fem.	*valeur* ‘value’	*valoir* ‘be worth’
-*eure*	*sculpteure* ‘sculptor’	*sculpter* ‘sculpt’
-*euse*	*tondeuse* ‘lawnmower’	*tondre* ‘mow’
-*in*	*pétrin* ‘dough trough’	*pétrir* ‘knead’
-*ine*	*accélérine* ‘accelerin’	*accélérer* ‘accelerate’
-*ing*	*kidnapping* ‘kidnapping’	*kidnapper* ‘kidnap’
-*ion*	*renonciation* ‘renunciation’	*renoncer* ‘renounce’
-*is*	*roucoulis* ‘cooing’	*roucouler* ‘coo’
-*ise*	*convoitise* ‘covetousness’	*convoiter* ‘covet’
-*isme*	*arrivisme* ‘ambitiousness’	*arriver* ‘succeed’
-*iste* masc.	*exorciste* ‘exorcist’	*exorciser* ‘exorcize’
-*iste* fem.	*séparatiste* ‘separatist’	*séparer* ‘separate’
-*ment*	*grondement* ‘rumble’	*gronder* ‘rumble’
-*oir*/-*oire*	*abattoir* ‘slaughterhouse’	*abattre* ‘slaughter’
-*oire*	*échappatoire* ‘way out’	*échapper* ‘elude’
-*on*	*forgeron* ‘blacksmith’	*forger* ‘forge’
-*onne*	*matonne* ‘prison guard’	*mater* ‘observe’
-*ose*	*hallucinose* ‘pseudohallucination’	*halluciner* ‘hallucinate’
-*ot*	*brûlot* ‘fire ship’	*brûler* ‘set fire’
-*ote*/-*otte*	*parlote* ‘chitchat’	*parler* ‘talk’
-*rice*	*fondatrice* ‘founder’	*fonder* ‘found’
-*ure*	*gerçure* ‘chapping’	*gercer* ‘chap’

We also decided to include diminutive (e.g. *-ette*), non-productive (e.g. *-ain*) and borrowed (*-ing*) suffixes, although questions have been raised about their status as deverbal French suffixes. Our decision was motivated by the aim to achieve the most complete description possible, and was based on the possibility to analyze at least two nouns with each suffix as derived from verbs in contemporary French.

### Selection of deverbal nouns

Functions of suffixes can only be observed through their realization in the lexicon. In this regard, the best way to list all possible functions of French deverbal suffixes would obviously be to collect and examine all deverbal nouns, which is quite unfeasible. A more realistic option is to analyze a randomized sample of deverbal nouns, but with the important risk of having some infrequent functions not represented. In order to meet feasibility constraints and to guarantee the best possible coverage of semantic functions, we decided to collect and scrutinize all deverbal nouns mentioned in (i) general studies aiming at a complete description of the derivational system in French (Apothéloz, 2002; Dubois, 1962; Thiele, 1987) and (ii) dictionary entries dedicated to the selected suffixes in lexicographic resources, viz. *Le Trésor de la Langue Française informatisé* (ATILF - CNRS & Université de Lorraine), *Le Petit dictionnaire des suffixes du français* (Editions Le Robert) and *Le Robert méthodique* (Rey-Debove, 1985). Given that both kinds of resources seek to provide a comprehensive overview of affix usage in French, we expect that the examples collected represent most existing semantic functions of deverbal suffixes. Nouns recommended as synonyms and/or appearing in specialized studies were also added to ensure that we would obtain the most exhaustive inventory possible.

This sampling method is not without limitations. First, the frequency of the different semantic types observed in the sample cannot be used as an indication of the overall distribution of meanings among deverbal suffixes, most notably because of a potential over-representation of rare meanings. Second, the sample collected remains dependent on the arbitrary choices of the authors of the resources. Although we tried to overcome this issue by using a variety of resources, authors’ preferences can still bias the selection of examples (e.g. in the case of derivatives formed with paired feminine vs. masculine suffixes, the former being potentially under-represented). Third, the selected derivatives are lexicalized words and as such their semantic properties may depend not only on derivational semantics (i.e. the semantic correlates of word-formation processes), but also on contingent effects of diachronic evolution, onomasiological necessities and lexical competition. As a consequence, it may be difficult to clearly identify the semantic functions of suffixes, especially if it turns out that some semantic types observed are idiosyncratic among deverbal nouns. These drawbacks should be taken into consideration when analyzing the sample of derivatives we collected.

Crucially, as we shall see in Sect. 4, a systematic re-evaluation of all collected nouns had to be carried out on the basis of homogeneous criteria, since methods of analysis vary from one resource to another. Before proceeding with the semantic analysis, however, it was necessary to guarantee the deverbal character of the collected nouns through an initial screening. Three conditions were set in this respect. The first is that the noun should be possibly analyzed as a suffixed word. Nouns that may be considered as both suffixed or converted (such as *contestataire* ‘protester’, possibly derived through suffixation from the verb *contester* ‘protest’ or through conversion from the adjective *contestataire* ‘protesting’) were thus kept in our sample. The second condition is that the base of derivation should possibly be a verb. A noun such as *boxeur* ‘boxer’ was included in the list because it can be analyzed as derived from the noun *boxe* ‘boxing’ (as in *football* ‘football’ → *footballeur* ‘football player’) but also from the verb *boxer* ‘box’ (as in *souder* ‘weld’ → *soudeur* ‘welder’). The third and last condition is that the base and the derivative can be semantically related in synchrony. This condition leads to the exclusion of demotivated nouns like *couloir* ‘hallway’ (historically derived from *couler* ‘flow’).

Following the application of these conditions, 3,091 nouns were finally retained in our list. The distribution of these nouns across suffixes is highly variable, with an average of 67 nouns per suffix (*SD* = 90). The 5 suffixes with the largest number of derivatives in the sample are: -*eur* (masc.) (*n* = 428); -*ment* (329); -*ion* (253); -*euse* (207); and -*age* (176). The suffixes with the smallest numbers of derivatives in the sample are: -*ase* (*n* = 3); -*ose* (4); -*ail* (6); -*eur* (fem.) (7); and -*ain*, *-elle*, -*iste* (fem. and masc.) and *-ote* (8).

## Semantic description of deverbal nouns

In this section, we present the semantic principles used to analyze our sample of deverbal nouns. Ontological and relational classifications of deverbal nouns are first distinguished (4.1), then described in more detail (4.2 and 4.3). Finally, we address the issue of lexical ambiguity with regard to morphological analysis (4.4).

### Forms of classification

Various semantic classifications of deverbal nouns have been proposed in the literature (see e.g. Fradin, 2012 for French; Kawaletz & Plag, 2015 for English; Ježek, 2007; Melloni, 2011 for Italian). These classifications usually fail to distinguish between information about the nature of the referent (e.g. event or state) and information about the relation with the eventuality denoted by the base verb (e.g. patient or result), which can lead to confusion in the description of nominalizations. Ontological and relational semantic components of deverbal nouns are different in nature, compatible rather than mutually exclusive, and not related one-to-one. For example, a deverbal noun that denotes an artefact can express different relations with the eventuality denoted by the base verb, whereas a deverbal noun that refers to the result of an eventuality can denote different kinds of referents, as illustrated in (1) and (2).

1.
  - *bâtir* ‘build’ → *bâtiment* ‘building’   [artefact-result]
  - *raser* ‘shave’ → *rasoir* ‘razor’   [artefact-instrument]
  - *garer* ‘park’ → *garage* ‘garage’   [artefact-location]

(2)
  - *bâtir* ‘build’ → *bâtiment* ‘building’   [artefact-result]
  - *créer* ‘create’ → *créature* ‘creature’   [animate-result]
  - *énerver* ‘irritate’ → *énervement* ‘irritation’   [state-result]

In this study, we will carefully distinguish ontological types (i.e. related to the nature of the referents) and relational types (i.e. depending on the semantic relation with the base), and provide a double classification of deverbal nouns. This method should allow for a detailed investigation of which semantic components of deverbal nouns are determined by the semantics of the derivational process. Lists of ontological and relational types are succinctly described in the next two subsections. More information is provided in the annotation guide that was used in this study and that is available in the supplementary material of the article.

### Ontological types

14 simple ontological types are distinguished based on distributional tests proposed in the literature on French nominal semantics (Godard & Jayez, 1996; Flaux & Van de Velde, 2000; Huyghe, 2015). For example, we assume that nouns denote events if they can be used as the object of the verbs *effectuer* ‘perform’, *procéder à* ‘proceed’ and *accomplir* ‘carry out’, or as the subject of the verbs *se produire* ‘occur’ and *avoir lieu* ‘take place’ (3). Nouns denote artefacts if they can be the object of the verbs *fabriquer* ‘manufacture’, *déchirer* ‘tear’, *construire* ‘build’ or *confectionner* ‘craft’ followed by various complements characterizing them from a physical point of view (4). More marginally, nouns are identified as denoting financial objects if they can be integrated into the expression *verser le N en euros* ‘pay the N in euros’ or *obtenir un N modique* ‘get a poor N’ (5).

(3)
  - Elle a procédé à une *réparation* difficile.^5^
    ‘She performed a difficult *repair*’
  - L’*éclosion* des oeufs a eu lieu dans l’après-midi.
    ‘The *hatching* of the eggs took place in the afternoon’

(4)
  - Elle a fabriqué une *génératrice* dans son garage.
    ‘She manufactured a *generator* in her garage’
  - Elle a déchiré l’*ordonnance* ce matin.
    ‘She tore up the *prescription* this morning’
  - Ils ont construit des *logements* gris en béton.
    ‘They built gray *dwellings* in concrete’
  - Il a confectionné des *roulades* au fromage.
    ‘He made cheese *rolls*’

(5)
  - Elle a versé la *redevance* en euros.
    ‘She paid the *license-fee* in euros’
  - Il a obtenu un *rendement* modique.
    ‘He got a poor *return*’

In order to provide accurate classification, the different tests are applied to one noun meaning at a time following a decision tree presented in detail in Haas et al. (2022). Ambiguous nouns can be assigned different ontological types (e.g. animate and artefact for *tailleur* ‘tailor’/‘suit’). In addition to the 14 simple semantic types, 7 complex types are used to account for nouns with a hybrid semantic structure consisting in the combination of two simple types. Complex types can be identified through the grammatical construction of co-predication, where predicates of distinct semantic types are jointly applied to one argument without any zeugma effect (see e.g. Cruse, 1995; Pustejovsky, 1995; Murphy, 2021). In (6) for example, the noun *discussion* ‘discussion’ is used both with a predicate typical of events (*avoir lieu* ‘take place’) and with a predicate typical of cognitive contents (*porter sur* ‘focus on’), without the two meanings being mutually exclusive. *Discussion* ‘discussion’ is therefore assigned a cognitive.event complex type.

(6) La *discussion* qui a eu lieu hier à huis clos portait également sur les modalités de suivi, dans la perspective d’une action commune future. (web)
  ‘The *discussion* that took place yesterday behind closed doors also focused on the modalities of follow-up, with a view to future joint action’

The complete list of simple and complex semantic types is presented in Table 2. Finally, we added to the ontological classification the possibility of indicating whether a noun is collective, being assigned a plural reference in the singular form (for French specifically, see Lammert & Lecolle, 2014). Some examples of collective deverbal nouns are given in (7).

(7)
  - *assister* ‘attend’ → *assistance* ‘audience’   [animate.collective]
  - *naître* ‘be born’ → *naissain* ‘spawn’   [natural.collective]
  - *tuer* ‘kill’ → *tuerie* ‘massacre’   [event.collective]

**Table 2:** Ontological types

Simple ontological type	Abbreviation	Example
animate	anm	*collaboratrice* ‘colleague’
artefact	art	*bouilloire* ‘kettle’
cognitive	cog	*plaisanterie* ‘joke’
disease	dis	*pelade* ‘autoimmune alopecia’
domain	dom	*jardinage* ‘gardening’
event	evt	*accouchement* ‘labor’
financial	fin	*redevance* ‘license-fee’
institution	ist	*association* ‘association’
natural	nat	*nageoire* ‘fin’
phenomenon	phn	*senteur* ‘scent’
property	ppt	*persévérance* ‘perseverance’
quantity	qua	*métrage* ‘length’
state	sta	*agacement* ‘annoyance’
time	tim	*échéance* ‘due date’
Complex ontological type	Abbreviation	Example
artefact.institution	art.ist	*restaurant* ‘restaurant’
artefact.cognitive	art.cog	*circulaire* ‘memorandum’
cognitive.event	cog.evt	*témoignage* ‘testimony’
event.financial	evt.fin	*paiement* ‘payment’
event.natural	evt.nat	*inflammation* ‘inflammation’
event.phenomenon	evt.phn	*crissement* ‘squealing’
event.state	evt.sta	*emprisonnement* ‘imprisonment’

### Relational types

Relational types correspond to roles of participants in eventualities. Generally speaking, they are equivalent to semantic roles possibly assigned to verb arguments. In this study, we consider 18 relational types, adapted from existing works on semantic roles, specifically *VerbNet* (Kipper-Schuler, 2005) and *LIRICS* (Petukhova & Bunt, 2008). Relational types are identified on the basis of conformity to definitions rather than linguistic tests, and differ from ontological types in that respect. Definitions of relational types with examples are given in Table 3. In addition to roles commonly found in the literature (e.g. agent, experiencer, theme, location), we also include a relational type reserved for transposition, i.e. when the noun denotes roughly the same type of eventuality as the base verb. For the same suffix, a distinction can thus be made, for example, between a verb-noun pair like *licencier* ‘dismiss’ → *licenciement* ‘dismissal’, where the same kind of event is denoted by the verb and the noun, and a pair like *agacer* ‘annoy’ → *agacement* ‘annoyance’, where the noun denotes a resulting state with respect to the verb. Transposition, as a change of lexical class without semantic modification, has been discussed by Beard (1995), Spencer (2010) and ten Hacken (2015), among others, and Lieber (2015) has argued that very few affixes or morphological processes are in fact transpositional. We consider here transposition in a coarse-grained approach: In the case of verb-to-noun transposition, it is equivalent to the preservation of the aspectual feature of dynamicity between base and derivative. The label transposition therefore applies to dynamic and stative eventualities, as in (8) and (9), respectively.

(8)
  - Construire cette maison a pris du temps.
    ‘Building this house took time’
  - La *construction* de cette maison a pris du temps.
    ‘The *building* of this house took time’

(9)
  - Je n’aime pas que Pierre se méfie de moi.
    ‘I don’t like that Pierre distrusts me’
  - Je n’aime pas la *méfiance* de Pierre envers moi.
    ‘I don’t like Pierre’s *distrust* of me’

**Table 3:** Relational types

Relational type	Abbreviation	Definition	Example
agent	agt	Entity that brings about an event intentionally	*forger* ‘forge’ → *forgeron* ‘blacksmith’
beneficiary	ben	Entity that receives or is dispossessed of something, or that is advantaged or disadvantaged by an event or a state	*hériter* ‘inherit’ → *héritier* ‘heir’
cause	cau	Entity that initiates an eventuality (not necessarily intentionally), or is the reason why an eventuality occurs	*agglutiner* ‘agglutinate’ → *agglutinine* ‘agglutinin’
destination	des	Endpoint in a change of location	*cracher* ‘spit’ → *crachoir* ‘spittoon’
experiencer	exp	Entity that is in or enters a particular state in relation to a psychological, perceptive or physiological stimulation	*adorer* ‘admire’ → *adorateur* ‘admirer’
extent	ext	Extensive value related to an event, or measurable magnitude of a change of state or location	*contenir* ‘contain’ → *contenance* ‘capacity’
instrument	ins	Entity that is manipulated in order to perform an action	*arroser* ‘water’ → *arrosoir* ‘watering can’
location	loc	Entity that serves as a landmark to locate another entity or an event	*fumer* ‘smoke’ → *fumoir* ‘smoking room’
manner	man	The way an action is performed, or the intensity of a state	*prononcer* ‘pronounce’ → *prononciation* ‘pronunciation’
path	pth	Trajectory followed during a change of location	*dévier* ‘divert’ → *déviation* ‘detour’
patient	pat	Entity that undergoes a (potential) change of structure	*mourir* ‘die’ → *mourant* ‘dying person’
pivot	pvt	Entity that is attributed a property, or is in a non-stimulated condition	*posséder* ‘own’ → *possesseur* ‘owner’
result	res	Entity that is created through an event	*égratigner* ‘scratch’ → *égratignure* ‘scratch’
source	src	Starting point in a change of location	*goutter* ‘drip’ → *gouttière* ‘gutter’
stimulus	sti	Entity that causes a psychological, perceptive or physiological state	*emmerder* ‘bother’ → *emmerdement* ‘bother’
theme	thm	Entity that is in a certain location or changes location	*charger* ‘load’ → *chargement* ‘load’
topic	tpc	Entity that is a subject of thought, discussion or cognitive activity	*deviner* ‘guess’ → *devinette* ‘riddle’

### Lexical ambiguity

Ambiguity is ubiquitous in verb-to-noun derivation and crucial to affix polyfunctionality, as noted in Sect. 2.2. Different meanings of deverbal nouns were postulated for any change of (i) base verb, (ii) ontological type, or (iii) relational type. For example, we distinguished different meanings for the nouns (i) *balayage* ‘sweeping’/‘scanning’, derived from distinct meanings of *balayer* ‘sweep’/‘scan’; (ii) *ravitaillement* ‘resupplying’/‘supplies’, ambiguous between an event and an artefact reading; and (iii) *maquillage* ‘cosmetics’/‘makeup’, ambiguous between an instrument and a result reading.

At least three patterns of lexical ambiguity can be considered, depending on whether the different interpretations of a noun are derived from different verb meanings (10), from the same verb meaning (11), or result from a figurative extension of meaning (12).

(10) Multi-base ambiguity
  V1 → N1
  V2 → N2

(11) Single-base ambiguity
  V1 → N1
  V1 → N2

(12) Figurative ambiguity
  V1 → N1 → N2

The ambiguity of verbs can be identified through variation of lexical aspect, argument structure and semantic role assignment. Noun and verb meanings were paired on the principle of nearest semantic proximity, as in the case of *balayer* ‘sweep’/‘scan’ and *balayage* ‘sweeping’/‘scanning’ described above. Figurative extension is manifest when one meaning of the noun is not semantically related to the base verb. For instance, the noun *lacet* ‘shoelace’, derived from the verb *lacer* ‘lace’, has another meaning ‘zigzag’ that is hardly traceable to the verbal base, but metaphorical in essence. However, in most cases, it is difficult to distinguish with certainty between single-base and figurative ambiguities, especially if noun meanings are potentially related by metonymy (e.g. *équipement* ‘equipment’ could be analyzed as derived morphologically from *équiper* ‘equip’ or metonymically from *équipement* ‘equipping’). The possibility of a dual formation, in which morphological process and figurative extension converge as a double motivation for polysemy, should not be excluded either. In our analysis of deverbal nouns, we encoded by default any possible semantic association between a verb and a suffixed noun, with the objective of further investigating ambiguity patterns based on the observation of actual cases of polysemy and on the comparison with monosemous derivatives.

Following the semantic principles described above, all nouns that were collected at the previous stage were systematically analyzed by a single annotator who indicated for each nominal meaning the base verb, the ontological type and the relational type. Problematic cases were discussed with a second annotator. The method had already been tested in a previous study involving both authors of the present study as annotators (Huyghe et al., 2023) and using the same analysis criteria, with a substantial inter-annotator agreement.^6^

A total of 5,212 meanings were identified from the 3,091 nouns collected in the previous step (see Sect. 3.2). 132 meanings were then excluded because they were strictly figurative, i.e. unrelated to the base verb, and thus did not depend directly on affix polyfunctionality. The remaining 5,080 meanings are associated with 1,752 monosemous and 1,339 ambiguous nouns, among which 388 fit into a multi-base configuration of ambiguity, 721 fit into a single-base configuration, and 230 fit into a combination of both configurations.

## Distribution of functions among suffixes

In this section, we investigate the diversity of possible functions associated with French deverbal suffixes, independently of their frequency of instantiation. Although the number of items present in our sample may provide some clues about the realization frequency of the different functions (see Sect. 6), the sample was not obtained from a randomized selection, as it depends on the arbitrary quantity of examples chosen to illustrate the semantic functions of the suffixes in the resources we consulted. All statistical analyses presented in this section are thus based on the presence or absence of a given function for a suffix.^7^ Several issues related to affix polyfunctionality are successively addressed: the heterogeneity of suffixes with regard to polyfunctionality and the diversity of functions observed (5.1); positive and negative associations between functions (5.2); the hierarchization of functions in the derivational system (5.3); and finally, the semantic competition between deverbal suffixes (5.4).

### Suffix heterogeneity and functional variety

**Fig. 1:**
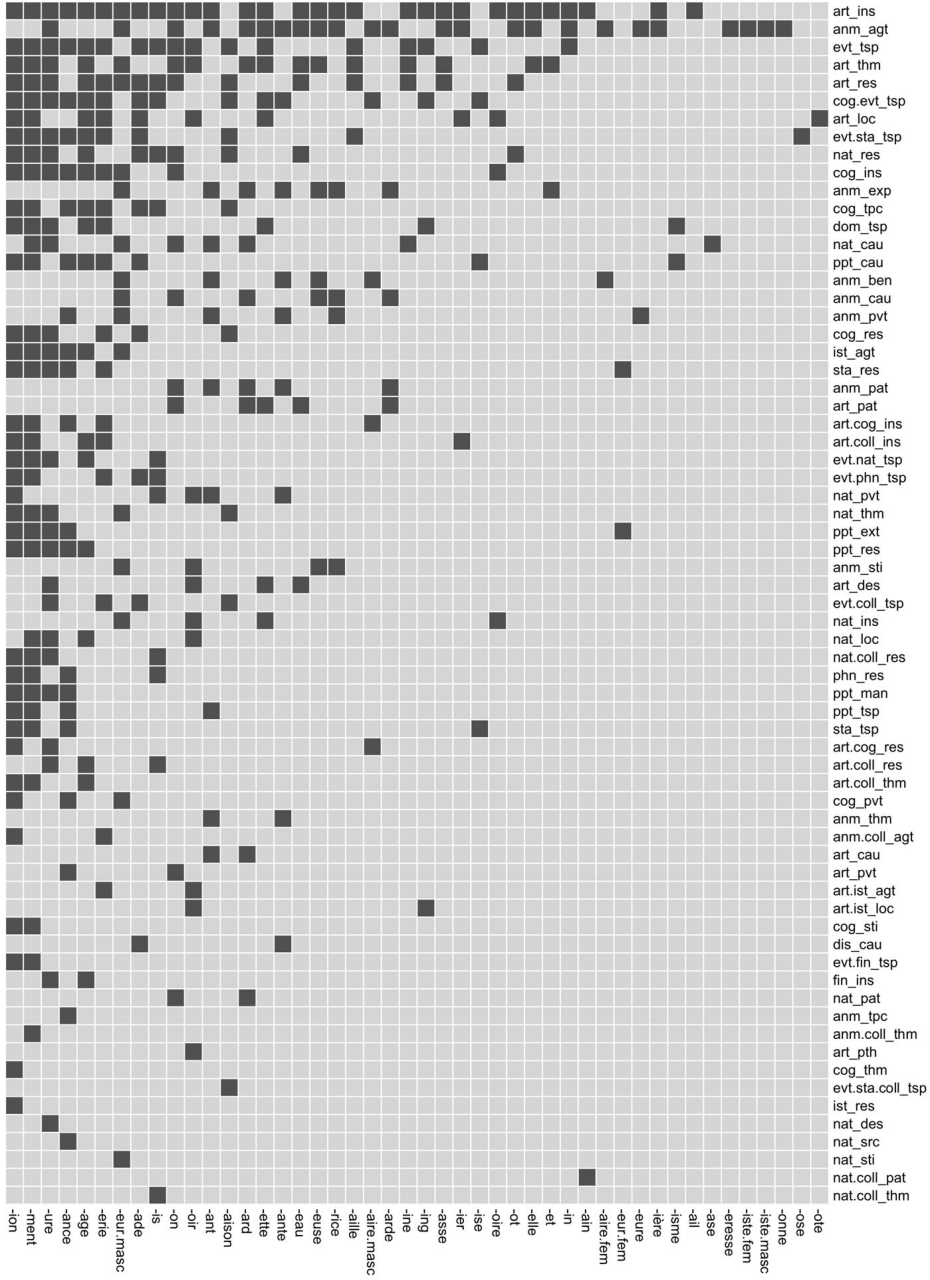
Combined functions per suffix. Functions are ordered from top to bottom, and suffixes from left to right, by decreasing frequency

In total, 101 combined semantic types (i.e. pairing ontological and relational types) were observed for the 5,080 meanings listed in our dataset. As indicated earlier, some of these types may be idiosyncratic and result from lexicalization effects such as semantic change and specialization, in which case they are not dependent on derivation itself. Consequently, they should not be taken into account when investigating the semantic properties of derivational processes. To control for the possible effects of lexicalization, we focused on cases where at least two derived words in our sample use the same suffix to realize the same semantic type. Such a selection increases the probability that the semantic types observed correspond to semantic functions of affixes strictly speaking, as opposed to independent lexicalized meanings. 347 lexical items (7% of the 5,080 meanings) for which the association of an ontological type and a relational type had only one instantiation per suffix were thus removed from our dataset. They corresponded to 34 semantic types, which brings the final number of combined functions to 67 (see Fig. 1 for the list of combined functions identified for each suffix).

Polyfunctionality is widely observed among deverbal suffixes, with an average of 8 combined functions per suffix (see Table 4), and only 8 monofunctional suffixes (-*ail*, -*ase*, -*eresse*, -*iste* masc., -*iste* fem., -*onne*, -*ose*, -*ote*). It is also quite variable. 5 suffixes are associated with 20 functions or more (*-ion*, *-ment*, *-ure*, *-ance* and *-age*, in descending order), whereas 22 suffixes are associated with less than 5 functions (e.g. *-elle*, *-eur* fem., *-in*, *-oire*, *-ot*). This variability may be related to differences in productivity, given that most weakly and highly polyfunctional suffixes seem to be weakly and highly productive deverbal suffixes, respectively. Counterexamples can probably be found (e.g. *-eur* masc. is presumably more productive than -*ance*, albeit less diverse, with 16 vs. 21 functions). Further exploration would be necessary to evaluate the exact relationship between polyfunctionality and productivity.

**Table 4:** Number of functions per suffix

	Range	Mean	Standard deviation
Ontological	[1,18]	4.7	4.7
Relational	[1,12]	4.6	3.2
Combined	[1,36]	8.0	8.4

Another observation concerns variation in polyfunctionality and gender alternation. As shown in Table 5, masculine suffixes are generally associated with a higher number of function than their corresponding feminine forms. It is unclear to what extent this stems from intrinsic differences or from sample biases attributable to the resources in which the nouns were collected. In the case of the denotation of animate entities, it seems likely that most of the functions identified for the masculine forms would also be identified for the feminine ones if a large corpus study was conducted. This result would be compatible with the proposal of Bonami and Boyé (2019), for whom gender-iconic pairs composed of masculine and feminine nouns that refer to males and females are related by inflection rather than derivation in French. On the other hand, irreducible differences between masculine and feminine forms seem to emerge when it comes to the denotation of inanimate entities, which justifies in this case the distinction between masculine and feminine derivational suffixes. For example, -*on*, unlike -*onne*, can form artefact-instrument nouns, as in *guidon* ‘handlebars’ and *pilon* ‘pestle’, whereas -*ante*, unlike -*ant*, can form cognitive.event-transposition nouns, as in *gueulante* ‘outburst’ and *beuglante* ‘rant’.

**Table 5:** Number of combined functions for gender-alternating suffixes

	Masculine	Feminine	Shared
-*aire*/-*aire*	5	2	2
-*ant*/-*ante*	11	9	7
-*ard*/-*arde*	10	5	5
-*eau*/-*elle*	7	3	3
-*et*/-*ette*	3	10	2
-*eur*/-*eresse*	16	1	1
-*eur*/-*eure*	16	2	2
-*eur*/-*euse*	16	7	7
-*eur*/-*rice*	16	6	6
-*ier*/-*ière*	4	2	2
-*in*/-*ine*	3	5	2
-*iste*/-*iste*	1	1	1
-*oir*/-*oire*	12	4	3
-*on*/-*onne*	13	1	1

Functions are numerous and frequently realized by several suffixes, with more than 5 suffixes per combined function on average (see Table 6). As before, considerable variation can be observed. Some functions appear to be extremely marginal, and 11 functions (i.e. 16% of all functions) are realized by one suffix only. Such is the case, for example, of animate-topic (realized by -*ance*, e.g. in *connaître* ‘know’ → *connaissance* ‘acquaintance’), natural.collective-patient (realized by -*ain*, e.g. in *couver* ‘brood’ → *couvain* ‘brood’), and institution-result (realized by -*ion*, e.g. in *s’associer* ‘join forces’ → *association* ‘society’). At the other end of the spectrum, 5 functions are associated with 15 suffixes or more: artefact-instrument (e.g. realized by -*eur* in *vaporiser* ‘spray’ → *vaporisateur* ‘sprayer’), animate-agent (e.g. realized by -*ard* in *piller* ‘loot’ → *pillard* ‘looter’), event-transposition (e.g. realized by -*age* in *atterrir* ‘land’ → *atterrissage* ‘landing’), artefact-theme (e.g. realized by *-eau* in *rouler* ‘roll’ → *rouleau* ‘rolling pin’), and artefact-result (e.g. realized by *-is* in *hacher* ‘mince’ → *hachis* ‘minced meat’), with 30, 24, 17, 16, and 15 suffixes, respectively. Interestingly, 4 out of these 5 most frequent functions consist in the denotation of concrete as opposed to abstract entities, i.e. objects as opposed to eventualities, which challenges common representations about the typical functions of nominalization.

**Table 6:** Number of suffixes per function

	Range	Mean	Standard deviation
Ontological	[1,33]	8.9	8.2
Relational	[1,31]	11.8	9.3
Combined	[1,30]	5.5	5.3

The relationship between ontological and relational types within functions can be examined as well. Not all possible combinations of both types are observed in our sample of deverbal nouns. Some ontological types are associated with many relational types (e.g. natural, artefact, animate combine with 10, 9 and 9 relational types, respectively), while others are associated with few relational types (e.g. event, domain and state combine with 1, 1 and 2 relational types, respectively). Conversely, some relational types are associated with many ontological types (e.g. transposition, result and theme combine with 11, 10 and 7 ontological types, respectively), while others are associated with few ontological types (e.g. beneficiary, manner and destination combine with 1, 1 and 2 ontological types, respectively). We do not find any reciprocal implication between ontological and relational types. However, some tight connections can be observed: property is the only possible ontological type for manner and extent, which are among the 5 possible relational functions for property; cause is the only possible relational type for disease, which is one of the 5 possible ontological functions for cause.

We used a chi-square test (with simulated values based on 2,000 replicates) to evaluate the dependence between ontological and relational types based on the combined functional types observed for each suffix. The test indicates a significant relation ($\chi ^{2} = 1047.8$, *p* < .001). A Cramér’s *V* measure of association between ontological and relational types, used to estimate the strength of the correlation, indicates a moderate dependence (Cramér’s *V* = .41). This result confirms that relational and ontological descriptions, although correlated to some extent, are not reducible to one another. Ontological types bring some semantic information that is not implied by relations. As a corollary, relational functions, although typical of derivational semantics, cannot fully account for it. The existence of regular and distinctive associations between ontological types and suffixes indicates that the semantics of verb-to-noun derivation is not restricted to relational information, but also includes ontological description.

### Associations between functions

In the sample of deverbal nouns we analyzed, some pairs of functions are realized more frequently than others by different suffixes. For example, event-transposition (*atterrissage* ‘landing’), artefact-result (*bâtiment* ‘building’) and animate-agent (*déménageur* ‘mover’) co-occur in pairs for a variable number of suffixes, as shown in Table 7. One may wonder whether semantic functions are randomly associated across suffixes or whether their co-occurrence is significant and possibly motivated.

**Table 7:** Number of suffixes for which pairs of functions are observed. The proportion of suffixes realizing a function (in rows) for which an association is observed is indicated in brackets

	event-transposition	artefact-result	animate-agent
event-transposition	-	11 (65%)	4 (24%)
artefact-result	11 (73%)	-	6 (40%)
animate-agent	4 (17%)	6 (25%)	-

To further analyze the associations between functions, we used a probabilistic model proposed by Veech (2013) and Griffith et al. (2016), originally intended to account for species co-occurrence across geographical sites in ecology. By drawing a parallel between species and functions, and sites and suffixes, this method allows us to estimate the probability that two functions would co-occur at a frequency different than observed if functions were independently distributed among suffixes. The model uses combinatorics and evaluates the number of ways that two functions can be distributed among suffixes to maintain the observed number of suffixes realizing each function. One can thus estimate the probability that two functions co-occur for a number of suffixes, ranging from 0 to the maximum possible number of suffixes, given the number of occurrences of the two functions among suffixes. The probability that the observed frequency of co-occurrence is significantly higher, lower or not different than expected can then be inferred. Accordingly, pairwise associations of functions can be classified as positive, negative or random.^8^

For example, in the case of event-transposition and artefact-result, with 17 and 15 suffixes realizing each function, respectively, the probability that both functions co-occur for a suffix is .121, and the expected number of suffixes realizing both functions is 5.5. In our data, 11 co-occurrences are actually observed. According to the model, the probability that a number of suffixes less than 11 would realize both functions is .99995, whereas the probability that a number of suffixes greater than 11 would realize both functions is .00062. These probabilities can be interpreted as *p*-values for negative and positive co-occurrence. At a significance level of .05, event-transposition and artefact-result can therefore be considered as positively associated in our data, i.e. significantly more frequently associated than expected if the 17 and 15 instances of each function were independently distributed among deverbal suffixes. The probabilistic evaluation of pairwise associations of event-transposition, artefact-result and animate-agent is presented in Table 8. It appears that event-transposition and animate-agent are negatively associated, since the probability of having a smaller number of suffixes than observed with both functions is lower than .05. artefact-result and animate-cause are randomly associated, since the probabilities of having a smaller or greater number of suffixes than observed with both functions are higher than .05.

**Table 8:** Probability of pairwise function associations. Information includes for each pair of functions the probability of co-occurrence, the number of expected and observed co-occurrences, and the probability that co-occurrence frequency would be lower or higher than observed if the two functions were distributed independently of one another

	Prob. cooc.	Exp. cooc.	Obs. cooc	Prob. lower	Prob. higher
evt-tsp/art-res	.121	5.5	11	.99995	.00062
evt-tsp/anm-agt	.193	8.9	4	.00342	.99960
anm-agt/art-res	.170	7.8	6	.20203	.92880

**Fig. 2:**
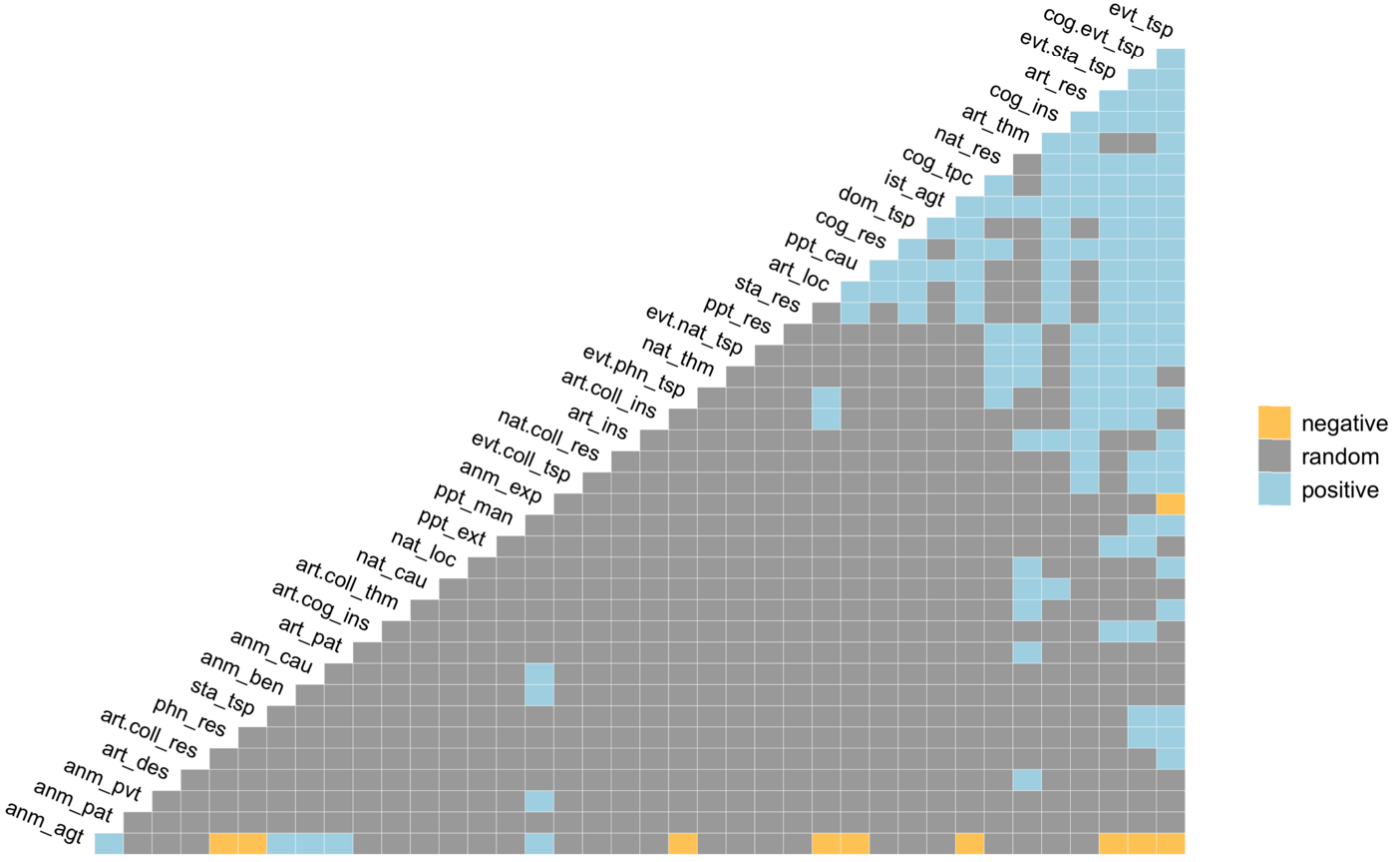
Positive, negative and random associations of functions. Functions without any significant negative or positive associations are trimmed out (*n* = 19). Functions are ordered from left to right, from the ones with the most negative associations to those with the most positive associations (Color figure online)

The probabilistic analysis can be extended to the 2,211 pairs formed by the 67 combined functions present in our data. As suggested by Veech (2013), a threshold is applied to eliminate functions for which the expected frequency of co-occurrence is less than 1, considering that these functions are not sufficiently represented in the data to allow for a reliable analysis.^9^ Consequently, 1,828 pairs (82.7%) were removed from the analysis. Among the 383 remaining pairs analyzed, 133 associations (34.7%) are positive, 10 (2.6%) are negative and 240 (62.7%) are random. As shown in Fig. 2, the 5 functions with the most positive interactions (range = [15,25]) are event-transposition, cognitive.event-transposition, event.state-transposition, artefact-result and cognitive-instrument. Although most of the pairs are random associations,^10^ an important number of functions co-occur among deverbal suffixes at a higher frequency than expected. By contrast, very few functions are negatively associated.

Positive associations between functions suggest motivation for co-occurring. The distribution of functions as being more or less frequently associated pairwise may be dependent on the semantic proximity between functions. It can be noted that many functions that are positively associated have semantic aspects in common, especially with respect to ontological description. Among the most frequently positively associated functions, we find those based on the denotation of events, artefacts and cognitive objects. At the other end of the spectrum, the functions with the fewest significant associations mainly involve the denotation of animates. Notably, animate-agent is the function with the most significant negative associations (*n* = 9). The description of similar relational types also seems to play a role in positive associations between functions, although to a lesser extent than ontological description.

If we analyze the distribution of pairwise associations between ontological functions only, out of the 123 associations with an expected frequency greater than 1 (45% of all associations), 69 (56%) are positive, 7 (6%) are negative and 47 (38%) are random. The five functions with the most positive interactions (range = [11,17]) are event, cognitive, cognitive.event, property, and event.state. All negative associations involve the animate function and indicate a clear separation in the semantics of deverbal suffixes between animates and eventualities (e.g. event, cognitive.event, property, event.state, domain). As far as ontological description is concerned, functions appear to be frequently associated positively, and the most associative functions are based on the event type. In contrast, out of the 99 associations for relational types with an expected frequency greater than 1 (65% of all associations), 26 (26%) are positive, none are negative and 73 (74%) are random. The relational functions with the highest number of positive associations (range = [5,6]) are transposition, result, cause, theme, and location.

These observations suggest that semantic information, in particular ontological description, could be a factor motivating associations between functions, thus contributing to the functional versatility of deverbal suffixes. We may hypothesize that polyfunctionality is determined, at least to some extent, by semantic relatedness between functions. In other words, part of the answer to why deverbal suffixes are polyfunctional and possibly associated with identical semantic functions may lie in the existence of conceptual relationships between functions, which encourages the assignment of multiple functions to a given suffix.

**Fig. 3:**
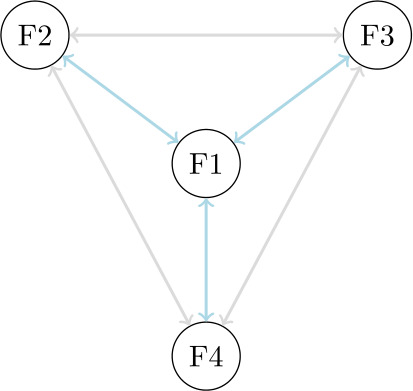
Radial organization of semantic functions. Direct and indirect semantic relations are indicated by arrows in blue and gray, respectively (Color figure online)

It remains that, according to the probabilistic analysis, random associations between functions are dominant. These associations are less easy to interpret than positive or negative associations in terms of semantic motivation. Functions that are randomly associated with respect to co-occurrence frequency may still be indirectly related semantically, if they are motivatedly associated with the same function. Accordingly, the semantic functions of an affix could be organized following radial structures, as schematized in Fig. 3. Out of the 240, 47 and 73 random associations observed for combined, ontological and relational semantic types, 90 (38%), 29 (62%) and 30 (41%) involve pairs of functions that are positively related to at least one identical function. Assuming that positive associations are semantically motivated, these results suggest that indirect semantic relatedness can be widespread in the organization of affix polyfunctionality. More extensive studies should analyze the nature of the functions that allow for indirect associations and determine their exact influence on the emergence of affix polyfunctionality.

### Hierarchy of functions

Among all the functions identified for a given suffix, some are only instantiated in our sample by ambiguous nouns related to a single base verb meaning. Despite sampling contingencies, this specificity suggests a hierarchy between primary functions, possibly realized in monosemous nouns, and secondary functions, realized only in ambiguous nouns.^11^ For example, event-transposition is a primary function for -*erie*, because it can be found in monosemous derivatives ending in -*erie* (13-a), whereas artefact-result is a secondary function for -*age*, because it only appears in ambiguous derivatives ending in -*age* (13-b).

(13)
  - *flânerie* ‘stroll’, *pillerie* ‘looting’, *tricherie* ‘cheating’
  - *collage* ‘gluing’/‘collage’, *moulage* ‘casting’/‘cast’, *tissage* ‘weaving’/‘woven piece’

**Fig. 4:**
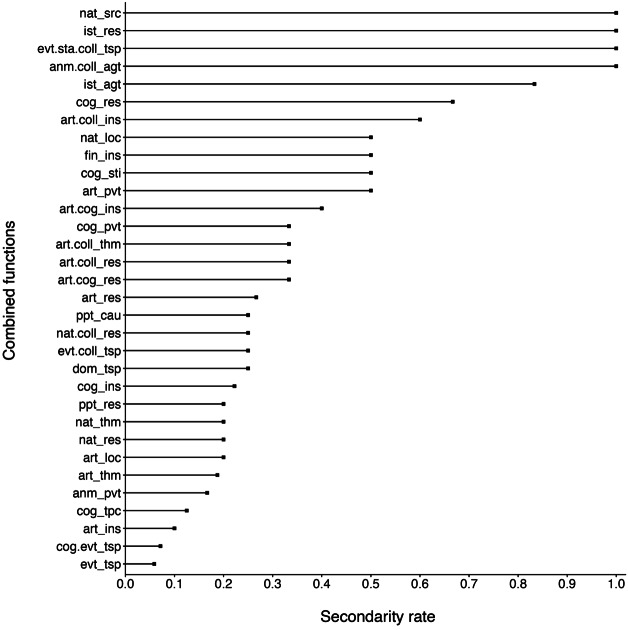
Rate of secondary realization of semantic functions. Only functions that are secondary for at least one suffix are represented

About half of the functions that we identified appear to be secondary for at least one suffix (*n* = 32/67). Figure 4 shows the list of these functions as well as their rate of secondary realization, which is calculated by dividing the number of suffixes for which a given function is secondary by the total number of suffixes that realize this function. Whereas functions with a low rate of secondary realization are necessarily instantiated by a substantial number of suffixes (e.g. event-transposition is realized as secondary by 1 out of 17 suffixes), functions with a high rate of secondary realization are generally instantiated by very few suffixes. For instance, the 4 functions that are always secondary in our data are realized by only 1 or 2 suffixes (e.g. *-ance* for natural-source as in *naissance* ‘birth’/‘base’, and *-ion* for animate.collective-agent as in *rébellion* ‘rebellion’/‘rebels’).

The hierarchization of functions calls for a reflection on the semantic operations associated with derivation. One may ask whether semantic types that are observed only in ambiguous nominalizations depend on derivational processes or result from independent patterns of semantic extension. A way to answer this question is to examine the meanings that co-occur in ambiguous nominalizations. If it appears that two semantic types are regularly associated in ambiguous derivatives and that the association is distinctive of a particular affix, then the formation of ambiguous words can be considered a property of the morphological process. For example, while the suffixes *-aire*, *-ant*, *-ard*, *-eur*, *-ier* and *-on* all realize the animate-agent function, only *-eur* can form ambiguous nouns with both an animate-agent and an institution-agent meaning, as in (14). Not only is institution-agent only realized for *-eur* in ambiguous derivatives with the animate-agent meaning, but also the association between these two meanings is only observed for *-eur*.

(14) *assureur* ‘insurance agent’/‘insurance company’, *distributeur* ‘distributor’/‘distributing firm’, *éditeur* ‘publisher’/‘publishing company’, *opérateur* ‘operator’/‘telecommunication company’, *transporteur* ‘carrier’/‘haulage company’

Similarly, unlike most eventive suffixes, *-ion* is regularly used to form nouns with both an event-transposition and an artefact-instrument meaning, as in (15). On the one hand, instrumental meanings with *-ion* are only observed for derived nouns that also have an eventive meaning. On the other hand, the formation of event nouns through suffixation does not systematically imply that of instrument nouns.

(15) *alimentation* ‘supply’/‘power supply’, *canalisation* ‘channeling’/‘pipe’, *climatisation* ‘air conditioning’/‘air conditioner’, *fixation* ‘fastening’/‘binding’, *séparation* ‘separation’/‘dividing wall’

It appears that even in the case of metonymic extensions, the ability to form words that are regularly polysemous between two given semantic types can be a distinctive feature of the affix. A possible theoretical explanation for this phenomenon is the existence of complex derivational patterns that include a regular polysemy component, and therefore allow for the formation of ambiguous derivatives with specifically related meanings (Salvadori & Huyghe, 2022).

### Polyfunctionality and rivalry

The sharing of functions between suffixes seems to be relatively common in the derivational system. In our sample, the average number of functions shared between pairs of suffixes is 1.7 (*SD* = 2.5, range = [0,30]). Important disparities can however be observed. For example, while *-ain* and *-isme*, *-ing* and *-eresse*, and *-oire* and *-onne* do not share any function, *-ion* and *-ment* have 30 functions in common (83% and 91% of their functions, respectively); *-ment* and *-ure* have 20 functions in common (61% and 74%); and *-ion* and *-ure* have 19 functions in common (53% and 70%). On average, each suffix shares 29% of its functions with other suffixes. Suffixes with the highest proportion of shared functions are *-ail* (64%), *-ière* (58%), *-eresse* (51%), *-iste* fem. (51%) and *-iste* masc. (51%), whereas *-eur* fem. (10%), *-ion* (13%), *-ance* (14%) and *-ment* (14%) are associated with the lowest percentages. It can be noted that the former are hardly polyfunctional and the latter highly polyfunctional (except for the suffix -*eur* fem.). There is indeed a negative correlation between the number of functions and the proportion of shared functions a suffix has (*r*(44) = –.54, *p* < .001).

Function sharing gives rise to situations of morphological competition. Arguably, a situation of rivalry between two affixes can be postulated if they have at least one function in common, which concerns 68% of all the pairs formed by the 46 suffixes. A certain gradience of the phenomenon should however be considered, depending on (i) the proportion of functions actually shared by the affixes, and (ii) the proportion of derivatives with shared functions. We take a closer look at (i) in this section and will further comment on (ii) in the next section.

**Fig. 5:**
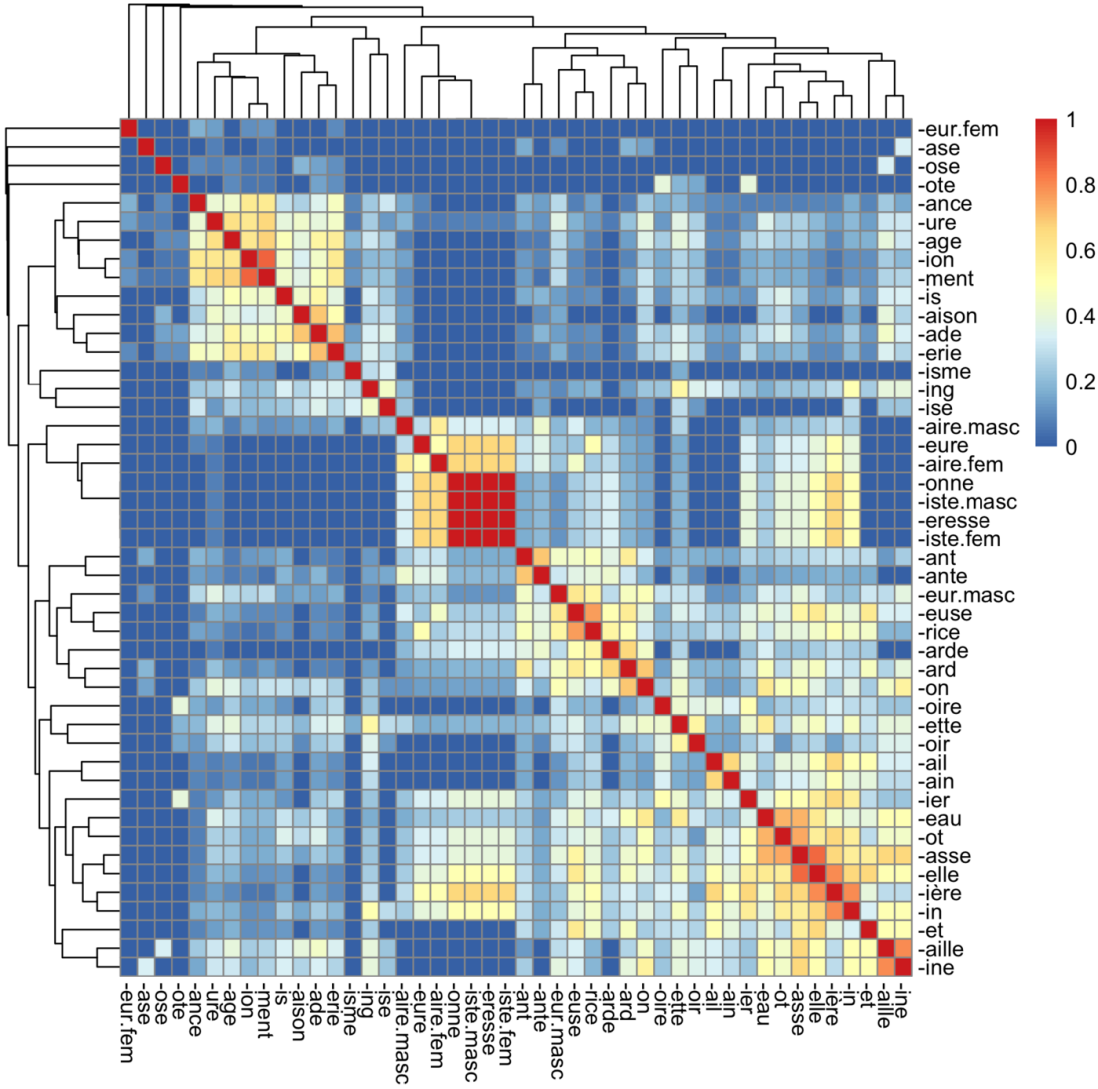
Heatmap representing the similarity between suffixes according to the functions they share. Warm colors indicate a strong similarity (Color figure online)

To determine the degree of rivalry between pairs of suffixes with respect to (i), we first built a suffix similarity matrix using Sørensen’s coefficient (Sørensen, 1948; Koleff et al., 2003; Legendre & Legendre, 2012), which is calculated according to the presence or absence of each function for each suffix.^12^ We then conducted a hierarchical clustering analysis to group suffixes showing similar behavior.^13^ Both analyses are presented in Fig. 5. While pairs of suffixes considered close according to Sørensen’s coefficient are displayed in warm colors, the dendrogram above and to the left of the heatmap reflects the result of the hierarchical clustering, as suffixes behaving in a similar way are grouped together. Using a cluster identification technique to find the best possible partition of the dendrogram,^14^ we finally distinguished 11 interpretable clusters of suffixes: 5 mini-clusters representing a single suffix (*-eur* fem., *-ase*, *-ose*, *-ote*, *-isme*), which are semantically isolated and hardly competing with other suffixes; 2 small clusters including 2 (*-ing*, *-ise*) and 3 (*-oire*, *-ette*, *-oir*) suffixes, respectively; and 4 larger clusters comprising between 7 and 12 suffixes. We retained the latter for further analysis (see Table 9 for the list of suffixes included in each of these 4 clusters). Given that they are associated with different degrees of intrinsic similarity and extrinsic contrast, not all groups are equally salient, as confirmed by the examination of the heatmap in Fig. 5. Groups A and B are more coherent and distinctive than Groups C and D, which are less clearly isolated from their neighbors. Group B appears to be particularly strong, perhaps because the suffixes that compose it present a relatively small number of functions.

**Table 9:** Main groups of rival suffixes as identified through hierarchical clustering. The order of presentation of the suffixes is the same as in Fig. 5

Group	Suffixes
A	*-ance*, *-ure*, *-age*, *-ion*, *-ment*, *-is*, *-aison*, *-ade*, *-erie*
B	*-aire* masc., *-eure*, *-aire* fem., *-onne*, *-iste* masc., *-eresse*, *iste* fem.
C	*-ant*, *-ante*, *-eur* masc., *-euse*, *-rice*, *-arde*, *-ard*, *-on*
D	*-ail*, *-ain*, *-ier*, *-eau*, *-ot*, *-asse*, *-elle*, *-ière*, *-in*, *-et*, *-aille*, *-ine*

In order to identify the semantic functions that are at the heart of these situations of rivalry, we estimated the importance of a function within a group of rival suffixes by dividing the number of suffixes realizing the function in the group by the number of suffixes composing the group. For example, the event-transposition function is realized by the 9 suffixes that compose Group A (realization rate = 1.00), but only by 1 suffix out of 8 (realization rate = .13) and 3 suffixes out of 12 (realization rate = .25) in Group C and D, respectively. Accordingly, this function can be considered distinctive of the rivalry within Group A. The realization rates of the main functions observed in each group of rival suffixes are presented in Table 10.

**Table 10:** Realization rates of functions eliciting competition within the 4 groups of rival suffixes. Only the functions for which half of the suffixes compete in at least one group are listed. Rates equal to or greater than .75 are indicated in bold. Functions that are not realized in a given group are represented by a dash

	Group A	Group B	Group C	Group D
animate-agent	.11	**1.00**	**1.00**	.58
animate-beneficiary	–	.29	.50	–
animate-cause	–	–	**.75**	–
animate-experiencer	–	–	**.88**	.08
animate-patient	–	–	.63	–
animate-pivot	.11	.14	.50	–
artefact-instrument	**.89**	–	**.75**	**1.00**
artefact-location	.56	–	–	.08
artefact-result	**.89**	–	.25	.42
artefact-theme	.44	–	.50	.50
cognitive-instrument	.67	–	.25	–
cognitive-result	.67	–	–	–
cognitive-topic	**.89**	–	–	–
cognitive.event-transposition	**1.00**	.14	.13	–
domain-transposition	.56	–	–	–
event-transposition	**1.00**	–	.13	.25
event.natural-transposition	.56	–	–	–
event.phenomenon-transposition	.56	–	–	–
event.state-transposition	**.89**	–	–	.08
institution-agent	.56	–	.13	–
natural-cause	.22	–	.50	.08
natural-result	**.78**	–	.13	.17
property-cause	.67	–	–	–
property-result	.56	–	–	–
state-result	.56	–	–	–

With regard to Group A, rivalry clearly hinges upon the denotation of events, artefacts and cognitive objects. The event-transposition, event.state-transposition, artefact-instrument, artefact-result and cognitive-topic functions are indeed associated with the highest rates in the cluster. Although the suffixes composing Group A are regularly referred to as “eventive” in the literature, these results indicate that their rivalry may be more extended than previously suggested. On the contrary, rivalry in Group B focuses on a single function: animate-agent. This is hardly surprising. Apart from *-aire* masc., which realizes 5 functions and is the most distant suffix in the cluster, suffixes in Group B are mainly monofunctional (*-iste* fem., *-iste* masc., *-onne*, *-eresse*) or bifunctional (*-eure*, *-aire* fem.). Most of them are feminine forms of gender-alternating suffixes (*-eure*, *-aire* fem., *-onne*, *-eresse*) that are associated with fewer functions than their masculine counterparts (see Table 5). While also competing for the animate-agent function, the suffixes making up Group C differ from those in Group B in that they rival for other functions involving animates (animate-cause, animate-experiencer), as well as for artefact-instrument. The latter is the main source of rivalry in Group D, whose most common functions are frequently observed in other groups and therefore not very distinctive. However, this group potentially differs from the others in two ways. On the one hand, given the diversity of the suffixes involved, it seems possible that in-group rivalry is here structured by family resemblances. Two given suffixes could be interconnected not because they have a lot of functions in common, but because their respective functions overlap with those of a third suffix that acts as an intermediary. On the other hand, we suspect the existence of additional meanings that would pertain to evaluative morphology, both measurative (e.g. *-eau*, *-ot*, *-et*) and appreciative (e.g. *-asse*). Although evaluative meanings are not directly captured by our annotation, they may be correlated to certain features we have encoded. It may be the case, for example, that some diminutives apply only to particular ontological types (e.g. artefact, animate), thus explaining part of the proximity observed in the clusters.

The existence of an apparently uneconomical derivational system that includes polyfunctional affixes with shared functions is intriguing.^15^ Admittedly, the semantic equivalence that defines affix rivalry can be evaluated at different levels of specification. A possible situation of rivalry may appear when considering coarse-grained semantic categories, but be excluded with a more fine-grained semantic classification. For example, deverbal suffixes competing for the event-transposition function could be differentiated when considering subclasses of events, based on aspectual differences (see e.g. Martin 2010). Nevertheless, one may surmise that, even when rival suffixes are associated with distinct fine-grained semantic functions, they also appear to be semantically undistinguishable in a number of derivatives (e.g. *rançonnage* and *rançonnement* ‘ransoming’, *finissage* and *finition* ‘finishing’).

Actual cases of rivalry observed in the lexicon may also be the result of diachronic evolution and past lexicalization (see e.g. Uth 2010). In this case, competing affixes are not necessarily any longer productive with respect to shared functions. Some situations of morphological competition, as in the third group of rival suffixes identified above, can thus be the remains of situations of rivalry that have been resolved historically (e.g. agentive *-on* and *-eur*), or the result of two affixes having accidentally the same functions at different periods.

In other cases, rivalry could be an effect of the semantic relationship between functions. As suggested in Sect. 5.2, polyfunctionality may be driven by conceptual associations between functions based on semantic relatedness and, as a result, may lead to situations in which different suffixes have identical functions. If both functions A and B are semantically related to function C, then affix *α* realizing A and affix *β* realizing B can be factors for *α* and *β* both realizing C. One can wonder whether the presence of some functions at the heart of the rivalry between deverbal suffixes would not reflect this phenomenon. Among the 10 functions with a realization rate equal to or greater than .75 in at least one of the clusters in Table 10, 6 can be found among the 8 functions with the most positive associations, as reported in Fig. 2. Conversely, the 10 functions associated with the highest number of positive associations all generate medium to strong competition, i.e. are associated with a realization rate equal to or greater than .50, in at least one of the clusters. This concomitance can be seen as an indication that some functions are widely shared because of their non-arbitrary co-occurrence among suffixes, and that the semantic associations between functions that drive polyfunctionality also cause affix rivalry. Affix rivalry could thus be considered an indirect effect of affix polyfunctionality, motivated by the underlying structures of the latter.

## Frequency of functions

Analyses in previous sections were all based on the inventory of the possible functions realized by deverbal suffixes. However, one may also wonder how often these functions actually occur in the lexicon. A quick survey of the 4,733 nominal meanings examined in Sect. 5 shows important disparities concerning their general frequency of instantiation. Some are largely over-represented, with animate-agent (*n* = 1154), event-transposition (*n* = 752) and artefact-instrument (*n* = 591) at the top of the list, whereas animate-topic (*connaître* ‘know’ → *connaissance* ‘acquaintance’), for example, appears only twice in the whole sample. Interestingly, disparities also emerge when one looks at one particular suffix. The artefact-instrument and artefact-location functions represent together 76% of the annotated meanings for *-oir*, whereas event-transposition (*parler* ‘talk’ → *parloir* ‘visit’) has only two occurrences. In contrast, the frequencies of the different instantiated functions seem a bit more balanced for *-ance*, as 7 functions (i.e. 33% of the functions identified for -*ance*) each represent more than 5% of the annotated meanings.

It remains that these preliminary observations may be biased, given that, as mentioned before, the sample of nouns we analyzed was not randomized but designed to represent the widest possible range of meanings. Providing a description of realization frequencies of all different functions for each suffix requires a very large amount of work, which is beyond the scope of our study. Nevertheless, on an exploratory basis, we examined a random sample of nouns derived with three suffixes: *-ade*, *-ment* and *-ure*. These suffixes were selected because they have a fairly high number of functions and were identified as rivals in Sect. 5.4, while still appearing in different subclusters. They should allow for further investigation of suffix polyfunctionality and rivalry when taking into consideration realization frequency. In this section, we present our methodology for drawing random samples of nouns ending with the three suffixes, and the results of their analysis with respect to polyfunctionality and morphological competition.

### Sampling and analysis

In order to evaluate the realization frequency of functions associated with *-ade*, *-ment* and *-ure*, a random sample of 100 deverbal nouns formed with each suffix was retrieved from the FRCOW16A corpus (Schäfer & Bildhauer, 2012; Schäfer, 2015), a French web corpus that contains 10.8 billion tokens. To obtain these 3 samples, the first step was to extract all lemmatized forms tagged as nouns and verbs from the corpus. We then automatically filtered verb-noun pairs that could be formally related (also taking into account patterns of allomorphy), which resulted in the selection of 292, 4,516 and 971 pairs for *-ade*, *-ment* and *-ure*, respectively. These pairs were randomly ordered, and the first 100 semantically related pairs for each suffix were retained. Note that the selected deverbal nouns are either lexicalized words, as listed in reference dictionaries, or neologisms (e.g. *se goinfrer* ‘pig out’ → *goinfrade* ‘pig-out’). Finally, we analyzed the semantic properties of the selected nouns according to the principles presented in Sect. 4. In total, 470 meanings were identified for the 300 nouns annotated: 145, 156 and 169 for *-ade*, *-ment* and *-ure*, respectively. To investigate the semantic aspects of derivation and to be consistent with the method adopted in Sect. 5, we considered as functions semantic types that are instantiated at least twice for a given suffix in the random sample, unless they have already been identified as functions of the suffix in the initial dataset. Following this procedure, we discarded 7, 3 and 2 meanings for -*ade*, -*ment* and -*ure*, respectively, which brings the total number of meanings under scrutiny to 458.

### Polyfunctionality and diversity

**Fig. 6:**
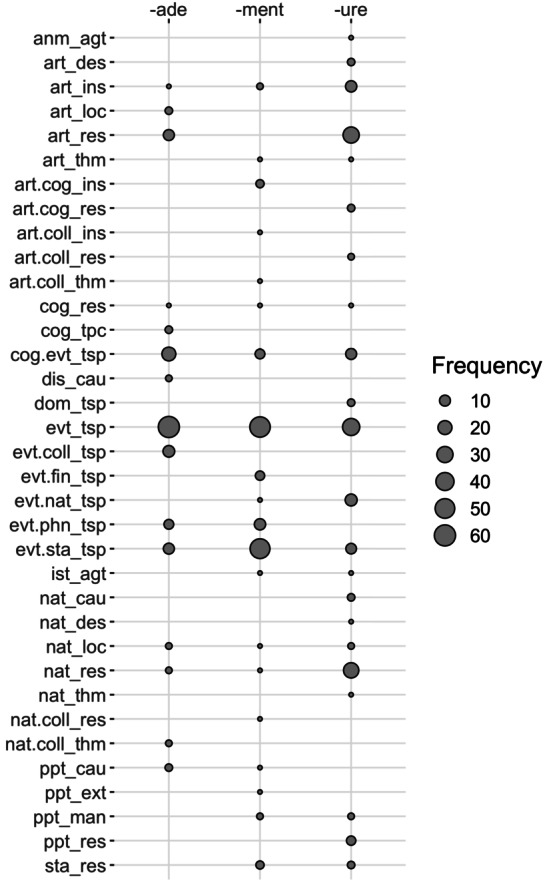
Frequency of functions per suffix

According to our semantic analysis, 35 different functions are realized in the random sample (15, 20 and 22 for *-ade*, *-ment* and *-ure*, respectively). As can be seen in Fig. 6, considerable differences in frequency can be observed between them. Covering alone 71% of the annotated meanings, the 5 most frequent functions are, in descending order, event-transposition (33% of the 458 meanings), event.state-transposition (15%), artefact-result (9%), cognitive.event-transposition (8%) and natural-result (6%). Transpositional events, possibly with a stative or a cognitive facet, and to a lesser extent resultative readings, are over-represented. By contrast, 22 functions account for less than 1% of the annotated meanings (e.g. animate-agent in *doubler* ‘understudy’ → *doublure* ‘understudy’). On average, a function has 13.1 lexical instances (*SD* = 27.8).

Some common tendencies can be observed when considering the frequency of functions per suffix, with the recurring dominance of event-transposition and the shared minority of cognitive-result and natural-location. Notable peculiarities also emerge for each suffix: -*ade* has the property of frequently forming event.collective-transposition nouns (e.g. *bousculer* ‘jostle’ → *bousculade* ‘melee’), whereas *-ment* forms almost as many nouns denoting events with a stative facet as nouns denoting pure events. As for *-ure*, it shows a predilection for nouns that denote concrete entities, with artefact-result (e.g. *enjoliver* ‘embellish’ → *enjolivure* ‘embellishment’) and natural-result (e.g. *écorcher* ‘graze’ → *écorchure* ‘graze’) meanings in particular.

The variation observed in the realization frequency of functions reveals differences in dominance between the different functions of a suffix. Affix polyfunctionality can be more or less balanced with respect to how functions are distributed among derivatives, which is an essential aspect of the semantic diversity of affixes. To evaluate both the functional richness of an affix and the evenness of distribution of its functions, we calculated for each suffix the Hill-Shannon index (Roswell et al., 2021),^16^ based on the number of functions associated with the suffix and the number of derivatives realizing each function. Commonly used in ecological studies to evaluate species diversity, this metric can also serve as a semantic diversity measure in morphology. The diversity index of an affix will be equivalent to the number of different functions the affix would have if these functions were perfectly evenly distributed among derivatives. In the random sample of deverbal nouns examined here, *-ure* (*D* = 11.6) appears as the most diverse suffix, followed by *-ade* (*D* = 7.0) and *-ment* (*D* = 6.4).

These observations confirm that the frequency of lexical realization is an important variable in the polyfunctionality of deverbal suffixes. It determines the weight of the different functions in the semantic profile of a suffix and consequently impacts the rivalry between suffixes.

### Competition

To assess the rivalry relationships between the three suffixes, we first calculated rates of co-functionality by dividing the number of common functions for a pair of suffixes by the number of functions that each suffix of the pair realizes. For example, *-ment* shares 9 functions with *-ade* and 12 functions with *-ure*, which represent 45% and 60% of its functions, respectively. As shown in Table 11, -*ment* and -*ure* are the suffixes that share the most functions, while -*ade* and -*ure* are the most distant. Co-functionality measures can be refined by taking into account the realization frequency of common functions. To do so, we divided the number of derivatives formed with a first suffix that instantiate the functions shared with a second suffix by the total number of derivatives formed with the first suffix. For example, *-ment* forms 130 derivatives that instantiate a function shared with *-ade*, which represents 85% of all the derivatives in -*ment* in our sample. In the case of *-ure*, 114 derivatives realize a function shared with *-ment*, which represents 68% of the *-ure* derivatives, as shown in Table 12.

**Table 11:** Number and proportion of shared functions (% per suffix in rows)

	*-ade*	*-ment*	*-ure*
*-ade*	-	9 (60%)	8 (53%)
*-ment*	9 (45%)	-	12 (60%)
*-ure*	8 (36%)	12 (55%)	-

**Table 12:** Number and proportion of derivatives instantiating shared functions (% per suffix in rows)

	*-ade*	*-ment*	*-ure*
*-ade*	-	106 (77%)	106 (77%)
*-ment*	130 (85%)	-	127 (83%)
*-ure*	124 (74%)	114 (68%)	-

A comparison of the results in Tables 11 and 12 provides a nuanced picture of rivalry. The proportions of derivatives with shared functions appear to be higher than the proportions of shared functions, showing stronger rivalry between the three suffixes. Furthermore, some proportions can be reversed. For example, -*ure* shares more functions with -*ment* than with -*ade*, but is actually used to form more derivatives instantiating a function shared with -*ade* than derivatives instantiating a function shared with -*ment*. Different aspects of rivalry are thus highlighted. Whereas the first results allow to detect cases of rivalry in terms of functions, the second ones can be used to weight the importance of the rivalry situations in which suffixes are involved.

To obtain a symmetric measure of rivalry that integrates function frequency and can be further generalized, we propose to quantify how similar two suffixes are based on their percentage difference distance, also known as the Bray-Curtis dissimilarity coefficient. By adapting this coefficient to evaluate similarity rather than dissimilarity, we obtain a measure that can be directly interpreted as a score of rivalry, ranging from 0 for no rivalry (i.e. complete dissimilarity) to 1 for absolute rivalry (i.e. identity).^17^ We calculated the similarity coefficients for the three pairs of suffixes observed, based on the semantic functions realized by their derivatives in our sample. The scores of rivalry obtained are, in descending order: .58 for *-ade*/*-ment*, .46 for *-ade*/*-ure* and .40 for *-ment*/*-ure*.^18^ Such measures make it possible to account for affix rivalry as a gradient relationship, depending on the polyfunctionality properties of affixes.

## Conclusion

In this article, we have studied the polyfunctionality of suffixes used to form deverbal nouns in French, for which a large-scale, systematic study was still lacking. More specifically, our aim was to find out (i) which semantic functions are realized by deverbal suffixes, (ii) how these functions are distributed in the derivational system, and (iii) whether there are motivated associations between them. To answer these questions, we collected a sample of 3,091 deverbal nouns ending with 46 different suffixes, drawn from studies aiming to exhaustively describe suffixation in French. In order to provide a homogeneous semantic description, each noun was reanalyzed using a double classification based on ontological and relational information.

Several characteristics of deverbal suffixes have been highlighted based on the examination of the sample. First, polyfunctionality appears to be widespread, as only a minority of the suffixes examined are monofunctional. Second, verb-to-noun derivation allows a very large variety of semantic functions, combining ontological and relational information. While some of them are very common among deverbal suffixes (e.g. animate-agent, artefact-instrument, event-transposition), others are more marginal (e.g. artefact-path, institution-result, natural-source). Third, functions are realized with very variable frequencies depending on the suffixes.

The study has also brought to light some fundamental aspects of affix polyfunctionality. Our results confirm that polyfunctionality can be driven by non-arbitrary semantic associations between functions, based on conceptual relationships between meanings, such as contiguity and analogy. Moreover, a hierarchy of functions can be postulated when considering the relationship between affix polyfunctionality and lexical ambiguity. The ability of affixes to realize functions through monosemous or ambiguous derivatives can be considered a third dimension to polyfunctionality, in addition to the number of functions they allow and the frequency with which these functions are instantiated. Finally, polyfunctionality appears to be inseparable from rivalry relations and to determine, through its structures, the degree of competition between affixes. Motivated associations between functions contribute to affix rivalry, which as a gradient phenomenon can be quantified based on the realization frequency of the functions common to different affixes.

More studies are needed to improve our understanding of affix polyfunctionality. Investigations should not be restricted to a certain morphosyntactic operation such as verb-to-noun derivation, but be extended to all affixes in a language, as well as to various languages. The abundance of functions and their distribution among types and tokens of derivatives should be carefully examined and evaluated through measures that take into account the different aspects of semantic diversity (Varvara et al., 2022). The relationship between affix polyfunctionality and lexical ambiguity also calls for further exploration, with respect to both interdependence and structural similarity. On the one hand, the influence of affix polyfunctionality on the formation of ambiguous words requires detailed quantitative analyses. The ability of polyfunctional affixes to form ambiguous derivatives may vary according to affixes and functions, and the co-occurrence of possible output meanings in derivatives should be scrutinized accordingly. On the other hand, the structures of affix polyfunctionality and lexical ambiguity can be compared, as both instantiate the one-to-many relation between form and meaning. One may wonder whether the motivation for semantic multiplicity and the relatedness between multiple meanings vary depending on the nature of the form. Differences between words and affixes in both the number of available forms and the possibility of coining new forms can influence homonymy rates and the relationship between the various meanings attached to the same form. As a consequence, different preferences could be observed between words and affixes in the organization of multiple meanings (e.g. more direct vs. indirect, or radial vs. vertical associations). From a general perspective, a structural comparison of affix polyfunctionality and lexical ambiguity may inform us on how language deals with meaning distribution in different types of ambiguous forms.

## Data Availability

The annotation guidelines, datasets and statistical script used in this study are available at https://github.com/deverbal-nouns/affix-polyfunctionality.

## Footnotes

1 This is not always the case. For example, Štekauer et al. (2012) report that in Gã, a Niger-Congo language, “each prefix and suffix has a single meaning, with the exception of the ... suffix *-mO*, which can form a gerundive but also derive an iterative/pluractional form of the verb” (p. 170).

2 An alternative term that could be used for “polyfunctionality” is “multifunctionality” (Luschützky & Rainer, 2013; Kenesei, 2014).

3 This is for example the point of view adopted by Olsen (2019) with regard to the agentive and instrumental functions of the English suffix *-er*. According to her, these functions are homonymous rather than polysemous, “in the same way that *bank* has two distinct meanings – the river bank and the financial institution” (p. 65). A similar suggestion is made by Luschützky (2011) about the semantic polyfunctionality of agentive suffixes in Slavic languages. According to Rainer (2014), “one could certainly make a case for treating English -*er*, Spanish -*dor*, Latin -*aculum*, and similar suffixes as homonymous rather than polysemous, at least in some of their uses” (p. 352).

4 There are a few masculine nouns ending in -*oire*, such as *conservatoire* ‘conservatory’ and *observatoire* ‘observatory’, which are similar to masculine nouns ending in -*oir* (e.g. in that they denote locations).

5 All deverbal nouns and their translations are in italics in the example sentences.

6 The observed inter-annotator agreement over 501 deverbal nouns was .83 for ontological types and .93 for relational types, corresponding to Cohen’s kappa values of .77 and .78, respectively, and prevalence-adjusted and bias-adjusted kappa (PABAK) values of .82 and .92, respectively.

7 Statistical analyses were performed in R version 4.2.1 (R Core Team, 2022). The following packages were used for specific tasks: Pheatmap (Kolde, 2019) for heatmaps and hierarchical clustering; cooccur (Griffith et al., 2016) for probabilistic functions co-occurrence; vegan (Oksanen et al., 2020) for dissimilarity indices; MeanRarity (Roswell & Dushoff, 2022) for Hill diversity.

8 The formula in 1 is used to calculate the probability that two functions #1 and #2 co-occur for exactly *j* number of suffixes, where *j* = 1 to *N*_1_ suffixes, *N*_1_ = number of suffixes realizing function #1, *N*_2_ = number of suffixes realizing function #2, *N* = total number of suffixes that were surveyed (possibly realizing both functions), ${\binom{N_{1}{}}{j}}$ = number of ways of selecting *j* suffixes realizing function #1 given that there are *N*_1_ such suffixes, $\binom{N - N_{1}{}}{N_{2} - j}$ = number of ways of selecting *N*_2_ – *j* suffixes realizing function #2 but not function #1 given that there are *N* – *N*_1_ such suffixes, and ${\binom{N{}}{N_{2}}}$ = total number of ways that *N*_2_ number of suffixes could be obtained out of a total of *N* suffixes.

1 $$ Pj = \frac{\binom{N_{1}{}}{j}\times \binom{N - N_{1}{}}{N_{2} - j}}{\binom{N{}}{N_{2}}} $$

Note that (1) is identical to Fisher’s exact test. The originality of the method proposed by Veech (2013) is to calculate the probability of co-occurrence for all numbers of possible associations in a sample and, based on the results, to provide a probability that a number of associations observed is higher or lower than expected by chance, thus inferring a polarity of association.

9 For informative purposes, we still performed the analysis on the 2,211 pairs of functions. Results indicate 1,758 (79.5%) random associations, 443 (20.0%) positive associations and 10 (0.5%) negative associations.

10 According to Veech’s criteria, only pairs of functions that do not differ significantly from their expected number of associations by more or less than 10% of the total number of suffixes should be classified as ‘truly random’. All the non-positive and non-negative cases observed in our data were truly random according to these criteria. In other words, no unclassifiable pair of functions was reported in the analyses, neither for combined nor for ontological or relational functions.

11 The distinction made here between primary and secondary functions is different from the one suggested by Lieber (2016), which is based on frequency and on the predominance of readings among derivatives (see also Lieber & Plag, 2022).

12 The formula in (2) is used to gauge the similarity of two suffixes, where |*X*| = number of functions of suffix *j*, |*Y*| = number of functions of suffix *k*, and |*X*∩*Y*| = number of functions common to suffixes *j* and *k*.

2 $$ \mathit{Sor}_{jk} = \frac{2 \lvert X \cap Y \rvert}{\lvert X \rvert + \lvert Y \rvert} $$

13 We used the average linkage method. The analysis consisted in (i) assigning a single suffix per cluster, (ii) combining into a single cluster the two closest clusters according to the matrix, (iii) computing the average distance between each suffix of the new cluster and each suffix of previous clusters, (iv) repeating steps (ii) and (iii) until there was only one cluster left that included all suffixes.

14 As suggested by Borcard et al. (2018, p. 83-84), we calculated correlations between binary matrices computed from the dendrogram cut at various levels and the original dissimilarity matrix, before selecting the level where the correlation was the highest.

15 Many factors (e.g. phonological, syntactic, sociolinguistic) can differentiate competing affixes and play a crucial role in the resolution of rivalry situations. We focus here on the semantic aspects of rivalry, considered as the defining feature of affix rivalry. Accordingly, gradience in rivalry is apprehended as gradience in semantic similarity between affixes.

16 The formula in (3) is used to gauge the diversity of a given suffix, where *S* = number of functions in the sample and *p*_*i*_ = number of items realizing the function *i* divided by the total number of items in the sample. This measure is also defined as the exponential of Shannon entropy.

3 $$ D = e^{-\sum _{i=1}^{S}p_{i}\ln(p_{i})} $$

17 The equation (4) gives the similarity between suffixes *j* and *k*, where *N*$_{ij}$ = abundance of function *i* for suffix *j*, *N*$_{ik}$ = abundance of function *i* for suffix *k* and min(*N*$_{ij}$, *N*$_{ik}$) = minimum of *N*$_{ij}$ and *N*$_{ik}$.

4 $$ R_{jk} = \frac{2\sum _{i=1}^{p}\min\left ( N_{ij},N_{ik} \right )}{\sum _{i=1}^{p}\left ( N_{ij}+N_{ik} \right )} $$

18 To empirically test rivalry scores based on percentage difference distance, we extracted and analyzed an additional sample of 100 deverbal nouns formed with the suffix *-on*, which does not belong to the same cluster of rivalry as *-ade*, *-ment* and *-ure*. The similarity scores observed for the different suffix pairs are, in descending order: .58 for *-ade*/*-ment*; .46 for *-ade*/*-ure*; .40 for *-ment*/*-ure*; .35 for *-on*/*-ure*; .19 for *-ade*/*-on*; and .10 for *-ment*/*-on*. Although *-ure* and *-on* remain relatively close—which is consistent with the fact that they both quite frequently form nouns that denote concrete entities—, the low scores observed for *-ade*/-*on* and *-ment*/-*on* seem to validate the measure we propose.
